# Dissociative Amnesia and Dissociative Fugue in a 20-Year-Old Woman With Schizoaffective Disorder and Post-Traumatic Stress Disorder

**DOI:** 10.7759/cureus.8289

**Published:** 2020-05-26

**Authors:** Tobechukwu A Clouden

**Affiliations:** 1 Psychiatry, Riverview Medical Center, Red Bank, USA

**Keywords:** case report, dissociative amnesia, dissociative fugue, post-traumatic stress disorder, schizoaffective disorder, case study, borderline personality disorder, psychotic disorder, psychosis, psychogenic amnesia

## Abstract

Dissociative amnesia is memory loss that cannot be explained by a neurological abnormality or typical forgetfulness. It belongs to the rare class of psychiatric ailments known as dissociative disorders. It can be accompanied with dissociative fugue where the individual travels or wanders away from home. This is a case of dissociative amnesia and dissociative fugue in a 20-year-old woman with schizoaffective disorder and post-traumatic stress disorder (PTSD). Dissociative amnesia associated with dissociative fugue is an even more rare phenomenon. This case is unique in that the patient also suffered from schizoaffective disorder and it demonstrates how dissociative disorders can be comorbid with a psychotic disorder. The amnesia itself offered antipsychotic and mood-stabilizing properties as the loss of memory eliminated her psychosis and mood instability.

## Introduction

Dissociative amnesia falls under the umbrella of psychiatric dissociative disorders. Dissociative disorders are a rare class of psychiatric ailments that cling to heals of trauma and stressful events. The dissociation is the mind’s way of hiding the traumatic event from the individual’s consciousness. Dissociative amnesia is defined as the “inability to recall important autobiographical information [1].” It has a prevalence of 1.8%, is the most common of the dissociative disorders, and is often diagnosed within the ages of 20-40 years old [1]. The memory loss is retrograde, sudden, and cannot be explained by a neurological cause or being forgetful. It usually follows an extremely stressful experience and is often associated with child abuse (particularly sexual and physical), suicide attempts, interpersonal violence, victimization, and deliberate self-harm. What is even more perplexing is the individual is often unaware of the amnesia, “amnesia for their amnesia [1].” Individuals can lose minutes in time to memories related to half of their life [2]. Awareness of memory loss is brought to light when others present the missing information to the individual. The formation of new memories is usually not impeded [3].

Dissociative amnesia may be associated with dissociative fugue, a variant of the amnesia, where the individual has purposefully traveled or wandered away from home or workplace [1, 4-5]. It has a much lower prevalence of 0.2% and bears the same onset, risks, and recovery like dissociative amnesia [6]. Dissociative fugue can last for hours to months and there is a loss of memory during the fugue state. There is no amnesia of events prior to the event or impediment on the formation of new memories [3-4, 6].

It is important to rule out other disorders when making the diagnosis of dissociative amnesia. Persons with neurocognitive disorders, brain lesions, head injuries, seizures, and substance use can present with signs that appear similar [1]. Dissociative amnesia can present with comorbidities of post-traumatic stress disorder (PTSD), somatic symptom disorder, conversion disorder, and personality disorders. 

Psychotherapy is the essence of treatment. The return of memory can be just as sudden as its disappearance, but the spectrum of memory recovery can be minutes to years. The closer the amnesia is to the stressful event, the quicker it can resolve [7]. Initially the individual must be made to feel safe and reassured freedom from danger. There are attempts to refamiliarize the individual with personal memoirs, belongings, and people with whom they are familiar. Individual therapy can offer support and validation, but the clinician should be aware of re-traumatization [3, 7]. Group therapy, family therapy, and hypnosis are also beneficial in retrieving memories. Medication can also be used to reduce anxiety and depressive symptoms associated with amnesia.

## Case presentation

The patient is a 20-year-old single woman who was referred to the ER by her group home for altered mental status. The staff declared her missing when she did not return from work. When she met with the treatment team on the psychiatric unit, she offered a scattered story related to the events prior to her admission. The team asked for clarification and further explanation, but the following story was pieced together.

She aged out of her group home with her most recent birthday and was forced to leave. She was left homeless and living on the streets for months. She narrowly escaped being raped on one occasion and desperately needed a place to live. She was walking the streets one day hoping to meet someone who would take care of her. She eventually met a nice man who brought her to his home and stayed with him overnight. However, the next day he started using cocaine and she needed to leave immediately. She was not threatened or abused by the man but perceived the environment as unsafe given his substance use. She asked for the custody of his two-year-old daughter in fear of the environment. She starts referring to the toddler as if she is her own child. The man agreed to her taking the child without any questions. The patient and the toddler left to begin walking down the turnpike. Her mother later joined the journey despite the patient’s warning of the long walk ahead of them. Eventually, a police car stopped, and an officer started walking toward them. He asked them to get into the car and they drove to a strange house. The patient went into the house with the officer, leaving her mother and toddler in the car. A woman in the house offered her some pills which she and the officer insisted the patient to take. The officer eventually left and drove off with her mother and the two-year-old in the car. She developed severe abdominal discomfort after taking the pills and an emergency medical services (EMS) vehicle brought her to the hospital.

The patient had no memory of the information obtained from the group home while she was in the ER. She did not remember her group home, staff, current medications, deteriorating mental status, hospitalization five weeks ago, intensive outpatient program, place of employment, or anything else that occurred while living in the home. The more questions the team asked the more puzzled the patient became. 

Mental status examination demonstrated a well-groomed woman who appeared her stated age. Her hair was somewhat unkempt, but she was generally well-groomed. She made continual facial expressions of disbelief and confusion, maintained good impulse control, and displayed normal psychomotor activity. Her speech was over productive with normal volume, rhythm, and rate. Her mood was “okay” and affect was in full range. Her thought process was circumstantial. Her thought content was illogical only when discussing the history of present illness, otherwise linear. She denied hallucinations and no delusions were elicited. 

The patient was admitted to the hospital approximately a month prior and discharged on olanzapine. Subsequently, olanzapine was restarted in the ER and she reported no side effects. When learning ziprasidone was given to her in the home, she did not want to restart the medication due to the previous abdominal discomfort. 

The hospital internist did not find any physical abnormalities during exam. Laboratory data, including urine toxicology and blood alcohol level, were of no clinical significance. Computed axial tomography (CAT) scan of the head without contrast was benign. The consulting neurologist denied any acute findings and the electroencephalogram did not demonstrate any seizure activity. The patient received a diagnosis of dissociative amnesia with dissociative fugue. 

The hospital was flooded with calls from the patient’s case managers that offered further needed information. She had been residing in a group home for the past seven months. She had a history of schizoaffective disorder, borderline personality disorder, and PTSD. She had an extensive history of trauma and an extremely strained relationship with her mother as a result. She was compliant with her medications, an active participant in her intensive outpatient program, and a responsible employee. She served as a role model for the other residents and was thriving in all aspects of her life until she started to decompensate approximately six weeks prior. 

She was becoming extremely worried about her upcoming birthday and associated departure from the group home. She was constantly reassured of an appropriate transition, yet she was certain of homelessness. She started becoming paranoid and acting bizarre. She was often engaged in self-dialog, cursing at herself and speaking with a Spanish accent. She believed her belongings were being stolen and frequently screamed at the other residents. On the day the patient went missing she accused her roommate of theft which escalated to a physical altercation. She angrily left the home and went to work. She completed her shift and told her co-workers she was waiting for a ride, although she usually went home on her own. She remained outside until all the employees left. The patient never returned home. Early the next morning she was reported missing. A police officer found her wandering along the highway that morning approximately 10 miles from her place of employment. He brought her to the group home where she had no memory of the staff or facility. She hesitantly agreed to take her ziprasidone, complained of abdominal distress, and accused the staff of poisoning her. EMS was called, and she was brought to the hospital. During her next encounter with the psychiatrist she described her memory loss to be like a nightmare. She remained completely puzzled with the reports of the last seven months. She often displayed a look of complete disbelief each time the psychiatrist asked about or revealed more information related to that time. When asked about the ongoing conflict with her mother, she quickly shook her head in disagreement and reported her to be supportive. She was skeptical about going back to the facility as it was an unknown place to her. However, she agreed to return if the treatment team thought it to be appropriate. 

While on the unit she was calm, cooperative, and pleasant. She was compliant with her medications and participated in the group therapy and milieu of the unit. She was interactive with her peers and often spoke to her mother on the phone. There were no signs of mood instability or psychosis. The hospital staff reported her to be helpful, appropriate, and with good behavior control. The patient’s case workers and support staff were hesitant for her return given continued amnesia, the other residents’ fears, and previous psychosis and violence. They did not feel it was appropriate for her return with such memory loss. The case workers thought the amnesia itself is enough to have the patient transferred to a higher level of care or discharge to a more supervised facility. Their apprehensions and requests were understandable; but the patient was mentally stable and did not meet the criteria for commitment. They were educated, had all their concerns addressed, and agreed to take her home with a discharge meeting prior to her return. She was discharged to the group home and would return to her intensive outpatient program and monthly sessions with the consulting psychiatrist.

One week later, the psychiatrist called to learn that the patient had not recovered any parts of her memory but had a smooth transition back into the facility. She was doing well and getting along with the other residents. She even started working at her previous place of employment. Her employer was willing to retain her given her work history. The psychiatrist called again in six months and learned she had transitioned into her new apartment. Faint pieces of memory had returned but she remained generally amnestic to those seven months. She continued to remain compliant with her psychiatric treatment, works, and does well.

## Discussion

This case report substantiates the relationship between extremely stressful events and dissociative amnesia. The idea of departure from the facility was a stressor her mind could not handle. Despite the reassurance of appropriate acclimation to the next level of independence, she remained in disbelief. She predicted an inevitable tragedy of complete abandonment and homelessness. The stressor led to the eruption of psychosis, mood instability, and erasure of the facility all together. 

The patient’s fugue state was interrupted by the police officer, but she remained amnestic to the previous seven months. The case demonstrates how dissociative amnesia may occur with dissociative fugue. However, studying their co-occurrence is extremely challenging given the rarity of these disorders. Both disorders follow traumatic events but there are no known predictors for their co-existence. 

She also began to display dissociative identity like symptoms prior to amnesia. The patient had no known history of dissociative symptoms but started talking in a Spanish accent as if to have another identity. These symptoms were mixed in with her paranoia and hallucinations, so the true nature of this dissociation is hard to understand. The patient’s dissociative-like symptoms may have also served as a precursor to the actual amnesia and fugue event. Other studies have discussed the appearance of dissociative symptoms in the midst of a psychotic disorder [8-9]. Such appearances are linked to the individuals who also have a history of trauma [8-9]. However, the patient’s dissociative amnesia and fugue were distinct events with no psychotic symptoms present, contrary to the literature. 

The replacement of the amnestic events with the patient’s own narrative was another unique part of her presentation. She had confronted the stressors of homelessness and abandonment in her narrative. In her mind, her worst fear had already come into fruition which may have helped to extinguish her psychosis and mood instability. The team tried to explore the psychodynamic explanations behind the nice man, the toddler, and the mother characters. The patient had a long history of verbal abuse, physical trauma, and sexual molestation. This made her vulnerable to anyone who showed her some form of affection and kindness. We questioned if the man represented her father who abandoned her at an early age. We reviewed the theme of heroism when she saved the toddler from her father. We explored the idea that the little girl represented the patient who also needed to be rescued when young. Another thought was the patient may have identified with the little girl and the rescue was her altruistic response. The narrative also may have been a delusion given her diagnosis of schizoaffective disorder. The substitution of previous autobiographical information with new material could be an additional finding within the diagnosis of dissociative amnesia. 

The patient had a history of being abandoned by her caregivers, including her mother. However, in the narrative her mother joins her and the toddler in their aimless journey. We suspect that her participation in the walk represented the patient's desire to have her mother by her side and provide emotional support. She started communicating with her mother who was a known stressor to her and was unable to recall their ongoing conflict. These forgotten memories were not explored as the patient became emotionally distressed when asked details about their relationship. The question emerges as to how these memories were also forgotten. There is the possibility that these memories were replaced with other narratives as well enabling the patient’s mother to serve as a source of comfort.

## Conclusions

Prior to this case dissociative amnesia has not been diagnosed in the presence of a known psychotic disorder. There are no known studies that have reviewed these diagnoses as co-existing. We learn from this case that dissociative amnesia can occur with a comorbid psychotic illness. Trauma is a known predecessor to retrograde amnesia. However, another caveat of this case is the substituted narrative that replaced the lost memories. In the patient’s case the new filler material eliminated her psychosis and mood instability. Further studies could explore the addition of a narrative substitution in the presence of dissociative amnesia. In addition, future documentation could investigate how the narrative affects the patient’s mental status. 
